# Carbonic anhydrase XII inhibition overcomes P-glycoprotein-mediated drug resistance: a potential new combination therapy in cancer

**DOI:** 10.20517/cdr.2020.110

**Published:** 2021-06-19

**Authors:** Kathryn F. Tonissen, Sally-Ann Poulsen

**Affiliations:** ^1^Griffith Institute for Drug Discovery, Griffith University, Brisbane 4111, Australia.; ^2^School of Environment and Science, Griffith University, Brisbane 4111, Australia.

**Keywords:** Carbonic anhydrase XII, P-glycoprotein, drug resistance, tumor microenvironment, pH, hypoxia, inhibitor, chemotherapy

## Abstract

Intrinsic or acquired resistance to chemotherapy is a major hurdle in the treatment of cancer. One of the key mechanisms of resistance is the overexpression of the drug efflux transporter P-glycoprotein (Pgp). Pgp overexpression renders a large number of mechanistically unrelated chemotherapies ineffective. Targeting Pgp inhibition directly to overcome drug resistance, although conceptually and mechanistically attractive, has not translated to the clinic, in part because Pgp also has a critical protective function in many healthy tissues. It was recently discovered that carbonic anhydrase XII (CA XII), an enzyme associated with pH regulation in cancer, is co-expressed and co-located with Pgp in drug resistant cancer cells. CA XII is also upregulated by hypoxia, which is another microenvironmental factor that contributes to drug resistance. Here, we review findings that demonstrate modulation of CA XII may offer a promising new approach towards overcoming the longstanding hurdle of drug resistance and therapy failure against solid cancers. This review covers the use of CA XII inhibitors, both small molecule and antibody, in combination with chemotherapeutics that are substrates for Pgp. This combination therapy approach restores the efficacy of chemotherapy in resistant cells and offers a potential new therapeutic window to re-examine the targeting of Pgp as a safe, effective, and novel anticancer strategy.

## Introduction

Cancer cells are highly proliferative, but due to poor vasculature and other metabolic alterations these cells utilize alternate pathways for energy generation as compared to healthy cells, including the glycolytic pathway. This is termed the “Warburg effect” and leads to an altered microenvironment that includes an increased production of lactic acid (or H^+^), the principal end product of glycolysis^[1,2]^. While tumor acidosis is recognized as a driver of cancer, supporting invasion and metastases, a cellular buffering system is essential to “dampen” intracellular pH displacements in response to elevated lactic acid. Regulation of intracellular pH is critical because of an association among protonation state, tertiary structure, and functional output of cellular biomolecules, such as proteins. After initially responding to chemotherapy, many cancers inevitably develop drug resistance, after which treatment ceases to be effective and disease recurs and progresses^[3,4]^. The subpopulation of cancer cells that evade drug treatment have intrinsic phenotypic differences compared to drug sensitive cancer cells^[5]^. One of the key mechanisms of drug resistance is the overexpression of drug efflux transporters^[6-8]^ of which P-glycoprotein (Pgp), also known as multidrug resistance protein 1, is one of the most abundant. Overexpression of Pgp in cancers may occur intrinsically if the cancer originated from a cell type with high basal Pgp expression. Alternatively, Pgp can be upregulated after exposure of cancer cells to specific drugs^[9]^. The high levels of Pgp protein conveys resistance towards a large group of mechanistically unrelated chemotherapies, rendering these drugs ineffective^[4,10,11]^. Although the co-administration of a Pgp inhibitor (e.g., tariquidar) with a chemotherapy treatment regime can in principle overcome Pgp-mediated drug resistance, this approach has faltered in many clinical trials with severe off-target toxicity caused by inhibition of Pgp present in healthy tissues^[10,12,13]^. Following the discovery that carbonic anhydrase XII (CA XII), an enzyme that counters extracellular acidosis in hypoxic tumors, indirectly reduces the activity of Pgp in resistant cancer cells, a new concept of targeting the pH microenvironment via CA XII inhibition to overcome Pgp-mediated drug resistance has arisen^[14]^. The co-expression of CA XII and Pgp is closely linked with a drug resistant phenotype^[14]^, but notably the relationship does not occur in healthy cells, allowing a new strategy to selectivity target Pgp only in cancer cells.

## Carbonic anhydrases

**Figure 1:**
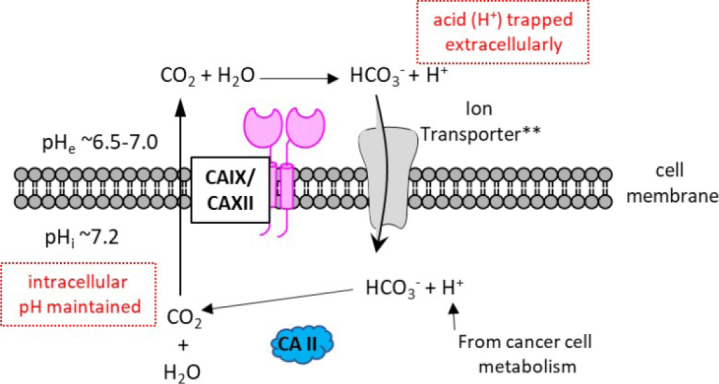
CAs IX and XII support pH homeostasis of the tumor microenvironment, involving both intracellular (pH_i_) and extracellular (pH_e_). CA: carbonic anhydrase, **HCO_3_^-^ is either co-transported with Na^+^ ions or exchanged for Cl^-^ ions (via anion exchangers).

**Figure 2:**
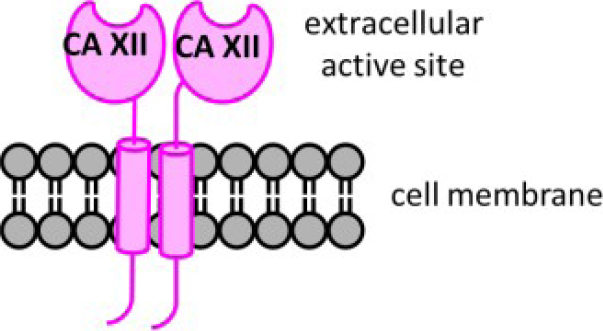
Schematic structure of CA XII, a membrane embedded dimer. Distinct features include: an extracellular active site domain (N-terminal), a short intracellular domain (C-terminal), and a single transmembrane spanning domain. CA: carbonic anhydrase.

In humans, there are 12 catalytically active carbonic anhydrase (CA, EC 4.2.1.1) enzymes. These enzymes have an active site zinc cation that provides an ideal environment to rapidly catalyze the reversible hydration of cell generated carbon dioxide (CO_2_) to bicarbonate (HCO_3_^-^) and a proton (H^+^) under physiological conditions^[15]^. There are two extracellular facing membrane-bound CAs, CAs IX and XII, which are induced by hypoxia and found highly expressed within the hypoxic core of many solid tumor types^[16]^. These CAs are expressed to a lesser extent in a variety of normal tissues^[17]^. Five CA isozymes are cytosolic (CAs I, II, III, VII, and XIII), two are in mitochondria (CAs VA and VB), one is secreted (CA VI), and two additional ones are membrane bound with an extracellular active site (CAs IV and XIV)^[15]^. CA isozymes differ in tissue distribution, subcellular location, abundance, enzymatic activity, and expression profiles in health versus disease, while all contribute to pH homeostasis across these settings^[15,18]^. CAs IX and XII support pH homeostasis of the tumor microenvironment, involving both intracellular and extracellular pH regulation [Figure 1]. They are implicated in tumor growth, metastasis, and progression, specifically attenuating the intracellular acid stress caused by the increased metabolism in cancer cells^[19-23]^. CA XII was first discovered in 1998^[24]^. It is a single-pass transmembrane protein, with a N-terminal extracellular catalytic domain, and a short, ~30 amino acid residue, intracellular C-terminal domain^[25]^. CA XII forms a dimer both in the crystal structure and in solution^[25]^ [Figure 2].

Intensive research has concentrated on the role of CA IX in hypoxic tumors, with CA XII less studied but also considered a driver in hypoxic tumors. CA XII was first identified in renal cell cancer as part of a study aiming to identify new tumor antigens via the application of SEREX, the serological identification of antigens by recombinant expression cloning^[24,26]^. The gene encoding CA XII, CA12, is a target of hypoxia-inducible factor 1 (HIF-1)^[21]^, and a common feature of many renal carcinomas is mutations in the von Hippel-Lindau (VHL) tumor suppressor gene, a regulator of HIF-1^[27]^. The VHL-encoded protein binds to HIF-1 resulting in its ubiquitin dependent degradation^[28]^. An early study showed that both CA9 and CA12 expression could be suppressed in renal carcinoma cell lines by expression of the wild-type VHL gene^[29]^. However, while subsequent studies attributed the high expression of CA IX in renal cancer to mutations in the VHL gene, CA XII expression only exhibited a strong trend and not an unequivocal relationship with VHL mutation status^[27]^. CA XII has since been found overexpressed in a range of human cancers including gastric, ovarian, lung, and brain^[29-38]^. In healthy cells, CA XII expression is low level and/or more restricted; it is found in the gastrointestinal tract, endometrium, pancreatic epithelium, and eye^[17]^, while CA IX is expressed in the stomach and peritoneal lining^[39,40]^. Unlike CA IX, CA XII expression is induced by estrogen^[32]^ and CA XII is highly expressed in estrogen receptor positive breast cancer^[32]^. Other studies have also shown differences between the expression and function of CA IX and CA XII in different cancers, including the involvement of CA XII in the promotion of tumor cell invasion and migration^[41-43]^. The role of CAXII in tumorigenesis may therefore depend on the cancer cell subtype, the hypoxic microenvironment, the progression of the disease, and to the more recently described drug resistant phenotype^[14,44-46]^.

### Small molecule inhibitors of carbonic anhydrase XII

Inhibitors of CA have been a pillar of human clinical intervention in diseases other than cancer for several decades^[47,48]^. Acetazolamide is a clinically used pan-CA inhibitor, approved for the treatment of epilepsy, glaucoma, edema, and acute mountain sickness, while also used for off-label indications^[49]^. Acetazolamide is one of the first reported CA inhibitors, discovered decades before either CA IX or XII were characterized. In addition to clinically used CA inhibitors, there is a vast number of small molecule CA inhibitors published^[15,50-52]^. These compounds encompass huge variation in activity, CA isozyme selectivity, and physicochemical properties. Since CA active sites are very similar, as evidenced by the many available Protein Data Bank structures of CA isozymes, it is challenging for medicinal chemists to specifically target a CA isozyme using rational drug design^[15]^. Fortunately, the marked differences in CA isozyme tissue distribution, subcellular localization, and expression in healthy versus diseased cells provide opportunities for medicinal chemistry programs to inhibit specific CA isozymes. The role of medicinal chemistry in the CA cancer field has thus been to develop small molecule inhibitors, not only with good enzyme inhibition activity but also with physicochemical properties that are useful *in vivo*, either as chemical probes or hit compounds for development into new CA-based cancer therapies. For example, medicinal chemists can fine-tune the physicochemical properties of small molecules to target a tissue or subcellular location selectively, or an extracellular facing enzyme, such as CA XII.

**Figure 3:**
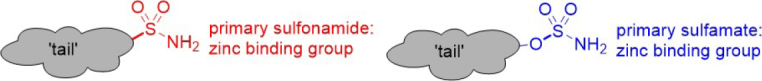
Generic structures of typical small molecule CA inhibitors. Distinct features include: (a) a zinc binding functional group to coordinate to the active site zinc cation; and (b) a “tail” group to interact with the CA active site and to optimize physicochemical properties. CA: carbonic anhydrase.

The recent focus of medicinal chemists has been on the discovery of inhibitors with specificity for the cancer-associated CA isozymes, CAs IX and XII. *In vitro* and *in vivo* studies in cancer models that directly investigate CA XII expression and inhibition are limited as compared to CA IX-focused ones. Many small molecule CA inhibitors have in common a zinc binding functional group [e.g., a primary sulfonamide (-SO_2_NH_2_) or primary sulfamate (-OSO_2_NH_2_)] in their structure [Figure 3]. This functional group is necessary to coordinate to the active site zinc cation and block the endogenous CA enzyme activity^[15]^. The remainder of the inhibitor structure, also known as the inhibitor “tail”, is developed for complementarity to the CA active site, to help to achieve isozyme specificity and for the incorporation of physicochemical properties that are compatible with drug discovery^[53-55]^ [Figure 3].

### Antibody inhibition of carbonic anhydrase XII

Zeidler and colleagues generated a specific monoclonal antibody that binds to and inhibits the catalytic domain of CA XII on tumor cells^[56]^. This antibody, designated 6A10, was generated following the immunization of LOU rats with A549 lung cancer cells in order to initiate the production of antibodies that recognize cell surface proteins. The team identified CA XII as the target of 6A10 and characterized the binding interaction as localized to the cell membrane of A549 cells. 6A10 was also shown to bind to a range of other CA XII expressing human cancer cell lines (gastric cancer, breast cancer, head and neck cancer, mesothelioma, and medulloblastoma), with a higher abundance of 6A10 binding observed at the site of cell-cell contacts, hypothesized as a more hypoxic microenvironment. In contrast, 6A10 does not bind to normal peripheral blood mononuclear cells, which lack CA XII. 6A10 was characterized as a potent inhibitor of recombinant CA XII activity (K_i_ = 3.1 nM) as determined using an assay that monitors the CA catalyzed reaction of hydration of carbon dioxide to form bicarbonate and a proton. 6A10 exhibited weak inhibition (K_i_ range 520-720 nM) of several other CA isozymes, namely CA VI (secreted), CA VII (cytosol), CA IX (transmembrane), CA XIII (cytosol), and CA XIV (transmembrane), with no inhibition of the remaining human CA isozymes (CAs I, II, III, IV, VA, and VB). This inhibition of CA XII activity contrasts with MAB2190, a commercially available CA XII antibody, which does not inhibit the activity of CA XII or other CA isozymes when using this assay^[56]^.

It is known that hypoxia is integral to the development of glioblastoma, a highly aggressive brain tumor type^[57]^. When human glioblastoma cell lines were cultured under hypoxic conditions, expression of CA XII was detected with 6A10. CA XII expression was not detectable when the same cell lines were cultured in normoxia. This finding is consistent with regulation of CA XII expression by HIF-1^[21]^ and the detection of CA XII in other tumor cells cultured in hypoxia^[56]^. Furthermore, when A549 lung cancer cells were grown in three-dimensional cell culture as multicellular spheroids, which display hypoxic characteristics, treatment with 6A10 reduced the growth of cells. The same cancer cells cultured under two-dimensional normoxic conditions were not inhibited by 6A10. Lastly, using a cell model that recapitulates cancer stem cells, CA XII was found highly expressed^[56]^. In a follow up study, Zeidler and colleagues showed that 6A10 exhibited antitumor activity in a xenograft lung carcinoma model, manifested through a signiﬁcant reduction of tumor load with concomitant increased overall survival^[56,58]^. The generation of a 6A10 antigen-binding fragment, designated Fab6A10, has recently been reported^[59]^. Similar to the parent antibody 6A10, the fragment selectively binds to and inhibits CA XII. In brain tissue samples, Fab6A10 specifically stains malignant glioma cells but not surrounding tissue. A crystal structure of a CA XII-Fab6A10 complex showed that Fab6A10 blocks the CA XII active site^[59]^. Although no *in vivo* work was reported, this study supports the therapeutic potential of targeting CA XII in cancer.

Moon and Ji disclosed in a recent patent application two therapeutic immunomodulatory CA XII antibodies. These antibodies, designated B4B and 27B6, have distinct and non-overlapping epitopes. They bind strongly to cell surface CA XII in a range of cancer cell lines *in vitro*, including lung adenocarcinoma A549 cells; colon LS147T cells; breast SK-BR3, MDA-MB-231, and MDA-MB-453 cells; and liver PLC/PRF5 cells^[60]^. It was shown that antibody binding does not affect cell viability and that the antibodies do not inhibit CA XII. When B4B and 27B6 were evaluated *in vivo*, using mouse models generated with MDA-MB-231 and MDA-MB-453 breast cancer cells, a reduction in solid tumor growth and tumor regression was observed. Notably, antibody 4B4 caused complete remission of MDA-MB-453 tumors in mice, with no tumor growth observed 21 days post-inoculation^[60]^. It was proposed that B4B and 27B6 act through the immune system, causing antibody-dependent cell-mediated cytotoxicity and complement-dependent cytotoxicity.

### Combination of CA XII inhibitor with Pgp substrate chemotherapies to overcome resistance

There is a small but growing number of preclinical and clinical studies exploring CA inhibition as a cancer therapy^[38]^. Those described herein have Pgp defined as a component of the mechanism of action. Of note, in the non-Pgp-related studies, many of which involve the pan-CA inhibitor acetazolamide, the CA inhibitor is commonly administered as a single entity and at a high concentration. In the preclinical studies reported herein, using co-administration of CA XII inhibitor with existing chemotherapies in Pgp^+ve^/CA XII^+ve^ cancer models, typically, cells are treated with CA XII inhibitor at nanomolar concentrations and mice are dosed with CA XII inhibitor at µg/kg levels.

Extracellular acidosis is an important characteristic of the tumor microenvironment that contributes to tumor progression, metastases, and stem-cell-like properties^[7,61-66]^. An additional and intriguing added complexity to this phenotype is the recent finding that CA XII is co-expressed and co-located with Pgp in drug resistant cancer cells^[3,14,44-46]^. Kopecka *et al.*^[14]^ applied cell surface capture technology, an unbiased method to perform a comparative quantitative analysis of the cell surface proteome. Cell surface capture technology selectively tags and purifies cell surface exposed glycopeptides for analysis by mass spectrometry. In this study, they characterized doxorubicin sensitive HT29 cells (Pgp^-ve^) and doxorubicin resistant HT29 cells, designated HT29/dx (Pgp^+ve^). The HT29/dx cells were prepared from parental HT29 cells using drug pressure, by multistep exposure to increasing concentrations of doxorubicin over 20 passages. HT29 and HT29/dx cells showed no difference in cell proliferation, spontaneous apoptotic cell death, or autophagy. One of the most notable differences observed between the proteomes was the finding that CA XII was upregulated (16-fold increased expression) in HT29/dx cells when compared to the parental HT29 cells. In addition to observing a progressive increase in Pgp and CA XII mRNA levels during the acquisition of chemoresistance, HIF-1a mRNA levels were also increased. Furthermore, the activity of HIF-1a, a regulator of CA XII gene expression, was stabilized in HT29/dx cells, even during normoxia^[14]^.

Using plasma-membrane extracts immunoprecipitated with anti-CA XII antibody followed by immunoblotting with an anti-Pgp antibody, it was found that CA XII and Pgp physically interact at the cell surface^[14]^. In a complementary experiment, plasma membrane extracts were immunoprecipitated with an anti-Pgp antibody and immunoblotted with an anti-CA XII antibody; again, a physical interaction between Pgp and CA XII was found^[14]^. This prompted the relationship between CA XII and Pgp to be characterized. When CA XII expression was silenced by specific siRNA, a decrease in ATPase activity of Pgp was observed and the HT29/dx cells were sensitized to the test Pgp substrates: doxorubicin (5 µM) or irinotecan (1 µM). The decrease in Pgp activity was caused by disruption of the optimal pH at which Pgp efflux occurs. A similar effect was found when HT29/dx cells were treated with acetazolamide (1 µM), a clinically used but nonspecific CA inhibitor^[14]^. The impact on doxorubicin intracellular accumulation was consistent with these findings, with HT29/dx cells accumulating significantly less doxorubicin than HT29 cells. However, when CA XII was silenced in HT29/dx cells, the level of intracellular doxorubicin reached the same level as measured in HT29 cells. Finally, the team also investigated the other cancer-associated CA, CA IX, in parallel. They found that CA IX played no role in the resensitization of resistant cells to Pgp substrates and that the mechanism was exclusively CA XII related^[14]^.

Synthetic and natural product CA XII inhibitors have recently been employed as chemical probes to explore the relationship between Pgp and CA XII in multidrug resistant cancer models, both *in vitro* and *in vivo*, as described in the next sections.

#### Glycoconjugate carbonic inhibitor study

**Figure 4:**
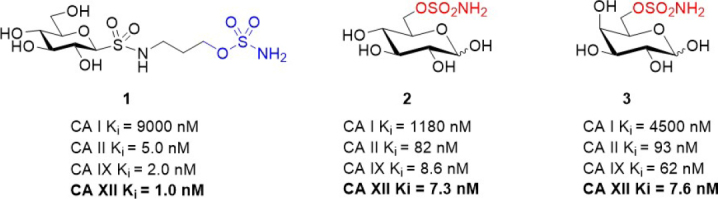
Glycoconjugate CA XII inhibitors that have been used in combination with Pgp substrates *in vitro* and *in vivo*^[44]^. CA: carbonic anhydrase; Pgp: p-glycoprotein.

Following the promising study described above, Poulsen and Riganti assessed a small panel of potent glycoconjugate CA XII inhibitors that were designed to target extracellular CAs *in vivo*^[67,68]^. The compounds were evaluated in a broad range of doxorubicin resistant cancer cell models that comprised drug sensitive parental cells and their resistant counterparts (colon, lung, breast, and bone)^[44]^. Resistant cells were generated by stepwise drug pressure selection in medium with increasing concentration of doxorubicin. The resistant cells express high levels of Pgp and CA XII, both of which were not detected in parental cells. Three structurally different CA XII inhibitors (sulfonamides or sulfamates) were identified that significantly increased the intracellular retention of doxorubicin to restore its cytotoxic activity to the level observed in drug sensitive parental cells [Figure 4]^[44]^. The CA XII inhibitors lower intracellular pH, which indirectly impaired Pgp ATPase activity, an index of Pgp activity associated with drug efflux action. Notably, the compounds exert their resensitization effect at a low concentration, 5 nM, whereas acetazolamide, a non-specific CA inhibitor, was effective at a 200-fold higher concentration (1 µM) in the initial study that characterized Pgp in concert with CA XII^[14]^. CA12-knockout assays confirmed that the drug resensitizing property of the compounds was dependent on CA XII enzyme activity interfering with optimal Pgp drug efflux^[44]^. Tariquidar, a direct Pgp inhibitor, was included as a control in the cell-based experiments. Tariquidar produces the same phenotypic response as indirect Pgp inhibition *via* CA XII inhibition. Compound 1 was selected for testing in a preclinical mouse model of drug-resistant breast tumors generated using mouse JC cells. Compound 1 has a K_i_ for CA XII inhibition of 1.0 nM. Based on its K_i_ value, it was evaluated *in vivo* at a concentration of 1.9 ng/kg and 1.9 µg/kg, with tariquidar (5 mg/kg) used as a control. Compound 1 at 1.9 µg/kg restored the efficacy of doxorubicin, a commonly used breast cancer drug, to the same extent as tariquidar^[44]^. This study provided further solid evidence that CA XII inhibitors, when used in combination with Pgp substrate drugs, overcome Pgp-mediated drug resistance in CA XII^+ve^/Pgp^+ve^ cancer cells, a typical phenotype of aggressive and drug resistant tumors of different tissue origins. Furthermore, CA IX was assessed in this study and ruled out as contributing to phenotypic changes associated with drug resensitization^[44]^. It is noted that many early studies on CA IX in cancer have not looked at a potential role for CA XII in parallel, and this impacts interpretation of findings.

#### Sulfocoumarin carbonic anhydrase inhibitor study

**Figure 5:**
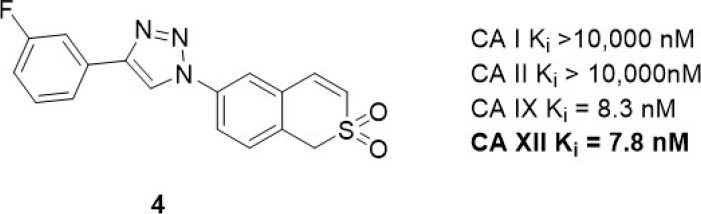
Sulfocoumarin CA XII inhibitor that has been used in combination with Pgp substrates *in vitro*. CA: carbonic anhydrase; Pgp: p-glycoprotein.

6-Triazolyl-substituted sulfocoumarins are an unusual class of CA inhibitor that show a selective inhibition profile against CAs IX and XII^[12,69]^. Similar to the closely related coumarins^[70]^, these compounds act as prodrugs. Specifically, they undergo hydrolysis to form a sulfonic acid functionalized compound, a group that may coordinate to the zinc-bound water molecule/hydroxide ion within the active site of CAs. These sulfocoumarins exhibit potent CA XII activity, with K_i_s in the low nanomolar range. Importantly, they are weak inhibitors against the abundant CA I and II isozymes (K_i_ > 10 µM)^[12]^, a profile indicating they are candidates for exploring the role of CA XII *in vitro*. A member of this inhibitor class, compound 4 [Figure 5], has been evaluated in multidrug resistant cell lines for non-small cell lung carcinoma, colorectal carcinoma, and glioblastoma alongside their corresponding drug sensitive cell lines^[67]^. *Pgp* expression was increased in the multidrug resistant cell lines. A significant difference in sensitivity between sensitive and multidrug resistant cells was observed only with non-small cell lung carcinoma (sensitive NCI-H460 and resistant NCI-H460/R) cells; this was however in the absence of chemotherapy, indicating that these compounds have a toxicity mechanism that may be unrelated to CA XII. Compound 4 sensitized multidrug resistant non-small cell lung carcinoma cells to doxorubicin and paclitaxel in resistant NCI-H460/R cells in a time (48-96 h) and concentration (0.5-2.5 µM) dependent manner^[69]^. The compound restored the intracellular level of doxorubicin in resistant NCI-H460/R cells to that in sensitive NCI-H460 cells. This effect was a response to indirect inhibition of Pgp activity as a result of CA XII inhibition disrupting pH. The reduction of Pgp activity was accompanied with increased Pgp expression, suggesting a feedback compensatory mechanism of multidrug resistant cancer cells.

**Figure 6:**
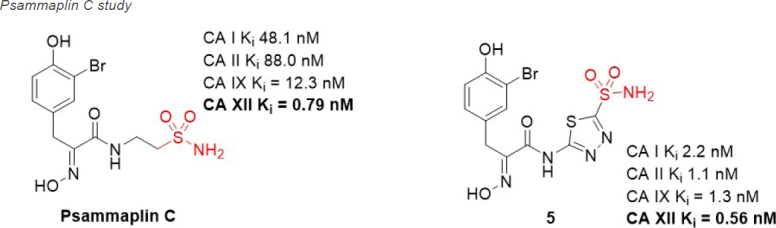
Psammaplin C, a natural product CA XII inhibitor that has been used in preclinical studies of glioblastoma in combination with Pgp substrate chemotherapeutics. CA: carbonic anhydrase; Pgp: p-glycoprotein.

Natural products (NPs) are compounds produced in nature that offer extraordinary chemical diversity. There is however a low representation of N-S bonds in nature^[71]^, and the classic CA binding chemotypes of primary sulfonamide and sulfamate are rare in NPs^[72]^. One notable NP sulfonamide, Psammaplin C [Figure 6], is a standout CA XII inhibitor (K_i_ = 0.79 nM), being one of the most potent CA XII inhibitors reported^[73,74]^.

**Figure 7:**
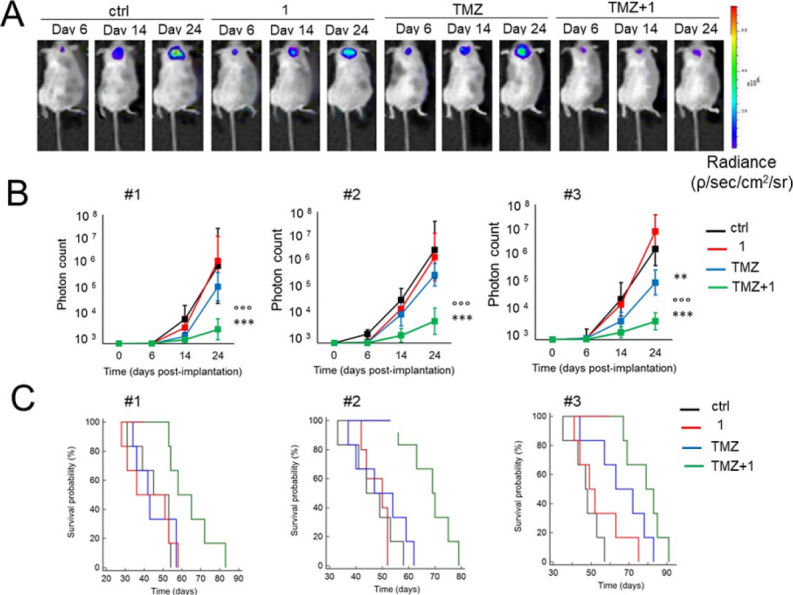
Psammaplin C (1) improves temozolomide efficacy against orthotopically implanted glioblastoma NS-derived tumor cells from three patients (#1, #2, and #3) into BALB/c *nu*/*nu* mice. (A) Representative *in vivo* bioluminescence imaging of orthotopically implanted Patient #2 NS, in animals treated with vehicle (ctrl), compound 1, and TMZ, as follows: (1) control group, treated with 0.2 mL saline solution intravenously (i.v.); (2) 1 group, treated with 3.8 µg/kg compound 1 i.v.; (3) TMZ group, treated with 50 mg/kg TMZ per os (p.o.); and (4) TMZ + 1 group, treated with 50 mg/kg TMZ p.o. + 3.8 µg/kg compound 1 i.v. (6 animals/group). (B) Quantification of Patient #1-3 NS-derived bioluminescence, taken as index of tumor growth. Data are presented as mean ± SD (6 animals/group). At Day 24: **P < 0.005 and ***P < 0.001, TMZ + 1 group *vs.* all the other groups of treatment; ^ooo^*P* < 0.01, TMZ + 1 group *vs.* TMZ group (Student *t*-test). (C) Overall survival probability was calculated using the Kaplan-Meier method. Patient #1 NS: *P* < 0.02, TMZ + 1 group *vs.* all the other groups of treatment. Patient #2 NS: *P* < 0.002, TMZ + 1 group *vs*. all the other groups of treatment. Patient #3 NS: *P* < 0.001, TMZ + 1 group *vs.* ctrl and 1 group; *P* < 0.05, TMZ + 1 group *vs.* TMZ group; *P* < 0.01, TMZ group *vs.* ctrl and 1 group (log-rank test; not reported in the figure). Figure adapted from^[46]^ and reused with permission. Copyright © 2018, American Association for Cancer Research. CA: carbonic anhydrase; TMZ: temozolomide; NS: neurosphere.

Glioblastoma is the most common, aggressive, and lethal adult primary brain tumor with a five-year survival rate well below 10%, underlining the seriousness of unmet medical need. A subpopulation of glioblastoma cells evade drug treatment, with resistance conferred by known genetic alterations including MGMT status, EGFR amplification, and mutations in IDH1/2 and TP53^[75]^. Until recently, the Pgp-CA XII relationship in glioblastoma was not known. Riganti and Poulsen investigated CA IX, CA XII, and Pgp expression in cell models from three different patient samples, with differing genetic signatures^[45,46]^. Patient cells were grown in 2D cell culture, as more differentiated adherent cells (AC), and in 3D cell culture, as stem-cell-like neurospheres (NS). The NS co-express CA IX, CA XII, and Pgp, while only CA IX expression is observed in AC. Confirmation that CA XII and Pgp are co-located in the cell membrane of NS was demonstrated using both a proximity ligation assay and co-immunoprecipitation methods. NS were resistant to temozolomide, a known Pgp substrate and the frontline chemotherapy in use for treating glioblastoma. In contrast, AC were sensitive to temozolomide. It was shown that CA XII activity mediates temozolomide resistance in a Pgp-dependent manner, with no identified role for CA IX in temozolomide resistance. Next, orthotopic xenografts derived from the temozolomide-resistant primary glioblastoma NS were prepared. Mice were resistant to temozolomide-only treatment (50 mg/kg); however, when mice were dosed with a combination of Psammaplin C (3.8 µg/mL) and temozolomide (50 mg/kg), the combination led to a significant increase in overall survival [Figure 7]. Treatment with the CA XII inhibitor alone had no effect and no detectable signs of systemic toxicity. This improvement in overall survival is unprecedented in preclinical studies of glioblastoma. The mechanism of action was characterized as CA XII inhibition indirectly reducing Pgp activity to not only restore temozolomide sensitivity but also reverse resistance to second-line chemotherapeutic substrates of Pgp^[76]^, topotecan, irinotecan, etoposide, and doxorubicin. In a follow up study, completed to build structure-activity relationships, 45 derivatives of Psammaplin C were designed, synthesized, and evaluated^[45]^. Compound 5 [Figure 6], a structural hybrid of acetazolamide and Psammaplin C, was a potent inhibitor of CA XII (K_i_ = 0.56 nM) and was investigated *in vitro* and *in vivo* as a combination therapy with temozolomide^[45]^. The *in vivo* study, orthotopically implanted patient-derived glioblastoma NS-derived tumors that co-express Pgp and CAXII into BALB/c nu/nu mice, resensitized drug resistant glioblastoma to temozolomide and showed a significant improvement in overall survival that was related to the CA XII mechanism of action to indirectly reduce Pgp activity in drug resistant cells^[45]^. The results of this second study, with the structurally different compound 5 that acts via the same mechanism, strengthen the possibility that co-therapy of temozolomide with a CA XII inhibitor may more effectively treat glioblastoma by suppressing this important temozolomide resistance mechanism.

### Inhibition of CA XII with antibodies to overcome metastases-a potential combination therapy

When Zeidler and colleagues characterized the CA XII-specific inhibitory antibody 6A10 in cancer cell models *in vitro* and *in vivo* (as described above), these studies were neither in combination with chemotherapy nor focused on drug resistance via Pgp^[56]^. Subsequently, the team demonstrated that 6A10, similarly to CA XII small molecule inhibitors, acts to indirectly reduce Pgp activity when applied to CA XII^+ve^/Pgp^+ve^ drug resistant cancer cells^[77]^. The effect of 6A10 co-treatment with Pgp substrates is to sensitize drug resistant cells to drugs that were ineffective when used alone. Co-treatment in *in vitro* models resulted in intracellular drug accumulation and subsequent cell death. However, unlike small molecule CA XII inhibitors, co-treatment of 6A10 with chemotherapy did not overcome resistance in a xenograft model generated with primary triple negative MDA-MB-231 breast cancer cells^[77]^. Doxorubicin was the drug used, a Pgp substrate used in the studies described with CA XII inhibitors. The authors noted that monoclonal antibodies are known to have limited effects where tissue penetration is poor as the case with many solid tumors^[78,79]^. A key finding however was that the combination therapy showed a significantly reduced number of metastases to the thorax when compared with animals treated with drug alone or 6A10 alone.

## Conclusion

Herein, we outline a novel method to inhibit Pgp indirectly and safely in cancer without off-target toxicity in healthy cells. The method is based on a recent discovery that CA XII, an enzyme associated with pH regulation in cancer, is co-expressed and co-located with Pgp in drug resistant cancer cells. It has been shown that a combination therapy protocol that combines a front-line chemotherapy that is a Pgp substrate together with a CA XII inhibitor blocks the Pgp-mediated resistance mechanism restores the efficacy of the chemotherapy in resistant cancer cells. This combination protocol has been demonstrated with multiple Pgp substrate chemotherapies and with multiple drug resistant cancer models, both *in vitro* and *in vivo*. Pursing CA XII inhibition as a future therapeutic option in drug resistant cancer is foreseeable, and it may overcome the long-standing difficulty of safely inhibiting Pgp drug efflux.

## Declarations

### Copyright

© The Author(s) 2021.
